# Supplementary material for: Complex‐centric proteome profiling by SEC‐SWATH‐MS
Source: Mol Syst Biol. 2019 Jan 14;15(1):e8438. doi: 10.15252/msb.20188438 (PMC6346213; doi:10.15252/msb.20188438)
Supplement: Supplementary file 7 — Dataset EV6 [file MSB-15-e8438-s007.zip › feature_plots_bioplex/P17026.pdf]

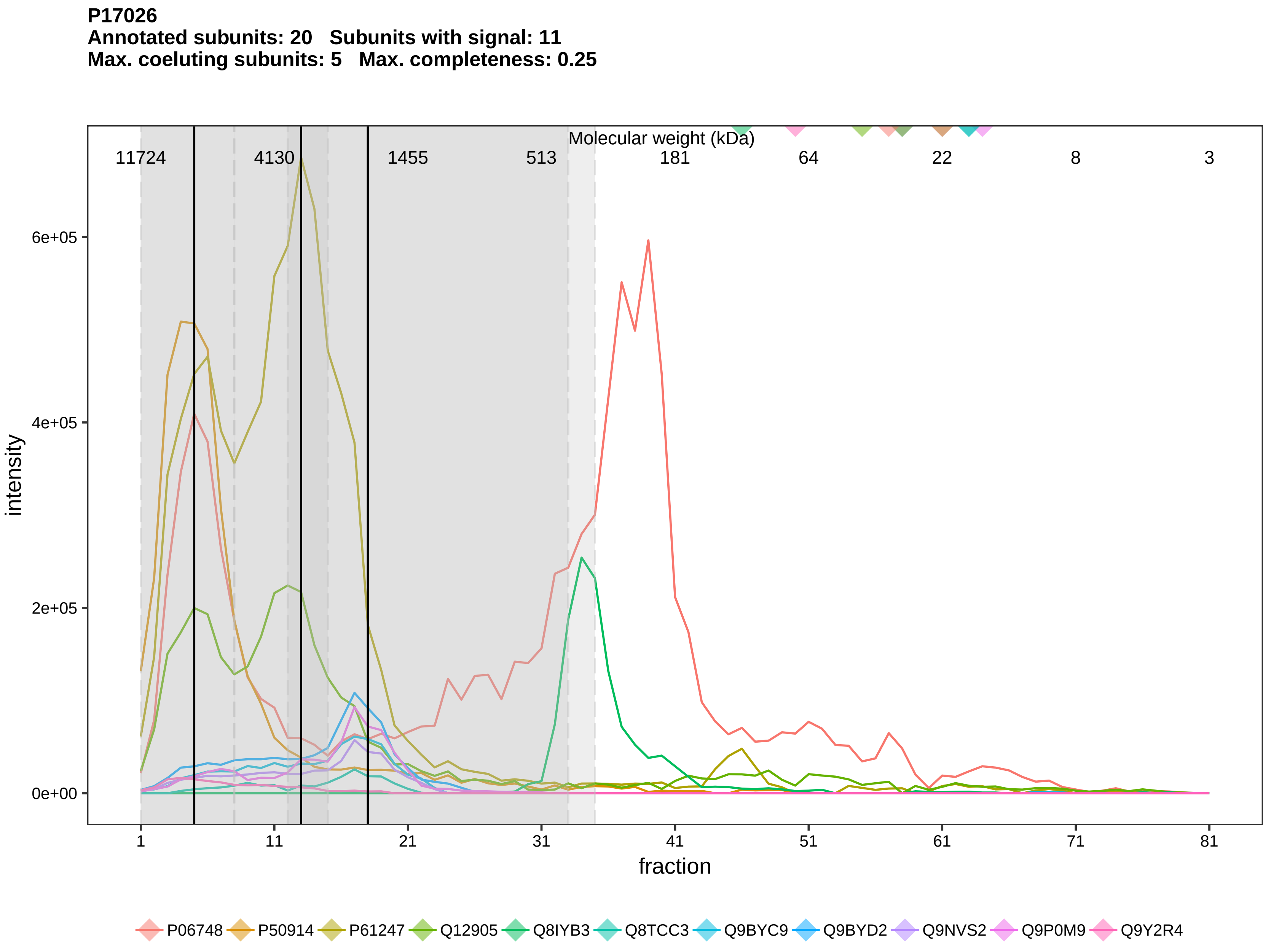
P17026
Annotated subunits: 20 Subunits with signal: 11
Max. coeluting subunits: 5 Max. completeness: 0.25
Molecular weight (kDa)
11724
4130
1455
513
181
64
22
8
3
intensity
6e+05
4e+05
2e+05
0e+00
fraction
1
11
21
31
41
51
61
71
81
P06748
P50914
P61247
Q12905
Q8IYB3
Q8TCC3
Q9BYC9
Q9BYD2
Q9NVS2
Q9P0M9
Q9Y2R4
